# Apoptotic cell clearance of *Leishmania major-*infected neutrophils by dendritic cells inhibits CD8^+^ T-cell priming *in vitro* by Mer tyrosine kinase-dependent signaling

**DOI:** 10.1038/cddis.2015.351

**Published:** 2015-12-10

**Authors:** F L Ribeiro-Gomes, A Romano, S Lee, E Roffê, N C Peters, A Debrabant, D Sacks

**Affiliations:** 1 Laboratory of Parasitic Diseases, National Institute of Allergy and Infectious Diseases, National Institutes of Health, Bethesda, MD, USA; 2 Laboratory of Molecular Immunology, National Institute of Allergy and Infectious Diseases, National Institutes of Health, Bethesda, MD, USA; 3 OBRR, CBER, U.S. Food and Drug Administration, Silver Spring, MD, USA

## Abstract

Neutrophils are the predominant recruited and infected cells during the early stages of *Leishmania major* infection in the skin, and depletion of neutrophils promotes immunity to infection transmitted by sand fly bite. In order to better understand how the acute neutrophilic response suppresses immunity, we assessed the consequences of the interaction between neutrophils recovered from the skin-inoculation site and bone marrow-derived dendritic cells (DCs) *in vitro*. The capture of infected, apoptotic neutrophils by the DCs completely inhibited their cross-presentation function that was dependent on engagement of the receptor tyrosine kinase Mer on the DCs. The capture of uninfected neutrophils, or neutrophils infected with *Toxoplasma gondii*, had only slight immunomodulatory effects. These studies define the clearance of infected, apoptotic neutrophils by DCs and Mer receptor signaling as central to the early immune evasion strategies of *L. major*, with relevance to other vector-borne pathogens delivered by bite to the skin.

The phagocytosis of dying cells in the absence of inflammation was described over 100 years ago by Metchnikoff in the context of the removal of regressing tissue during amphibian morphogenesis.^1, 2^ Many studies have since addressed how dying cells that are continuously generated as a consequence of normal tissue turnover signal for their clearance by dendritic cells (DCs) or macrophages in a manner that maintains homeostasis. Phagocytosis of apoptotic cells by macrophages and DCs can inhibit their production of proinflammatory mediators while leading to the production of TGF-*β* and to the generation of regulatory T cells.^3, 4, 5^ Under steady-state conditions, the capture of apoptotic cells by DCs is thought to contribute to the maintenance of peripheral tolerance.^6^ In infection-driven inflammatory settings, the ingestion of apoptotic cells by macrophages and DCs may be exploited by pathogens to promote infection by inhibiting components of the antimicrobial response. For example, *Trypanosoma cruzi* grows better in macrophages that have ingested apoptotic lymphocytes;^7^ the killing of *Streptococcus pneumonia* by alveolar macrophages is suppressed following their uptake of apoptotic cells;^8^ and *Mycobacterium tuberculosis*-induced activation of human DCs for T-cell priming is inhibited by their co-culture with apoptotic neutrophils.^9^

During infection, PMNs and DCs, which are normally located in distinct anatomical compartments, may colocalize at sites of inflammation. As neutrophils are short-lived cells that must be targeted for orderly removal, the function of DCs as antigen-presenting cells (APCs) in these infectious foci may become subordinate to their role in the clearance of dying neutrophils. For microbes that gain entry into the skin via the bite of an arthropod vector, the inflammatory signals that direct the recruitment and colocalization of neutrophils and DCs can be especially pronounced as they are driven not only by the microbial stimuli, but also by salivary constituents and by the tissue injury associated with the bite itself. In this context, we have previously reported that the inoculation of *Leishmania major* into the skin by sand fly bite or by needle results in the rapid recruitment of large numbers of neutrophils that phagocytose the parasite and constitute the predominant initial infected cell in the site.^10, 11, 12^ Although the majority of the infected neutrophils die and release viable parasites that are taken up by inflammatory monocytes in the skin, a significant proportion appear to be engulfed by DCs, with most infected DCs acquiring their early infections via this process.^11^ As neutrophil depletion just before infection augments the development of immunity to sand fly-transmitted infection and of a *Leishmania*-specific T-cell response, the capture of infected neutrophils by DCs in the skin was suggested as a key mechanism to inhibit their APC function and to delay the development of acquired resistance. The current studies were designed to model these cellular interactions *in vitro*, and to directly explore the immunologic consequences of the capture of *L. major-*infected neutrophils by DCs. The findings reveal a profound impairment of the capacity of DCs for T-cell priming following their uptake of infected neutrophils that is mediated by Mer tyrosine kinase-dependent signaling.

## Results

### DCs capture skin-recovered *L. major*-infected and uninfected neutrophils *in vitro*

**Figure 1:**
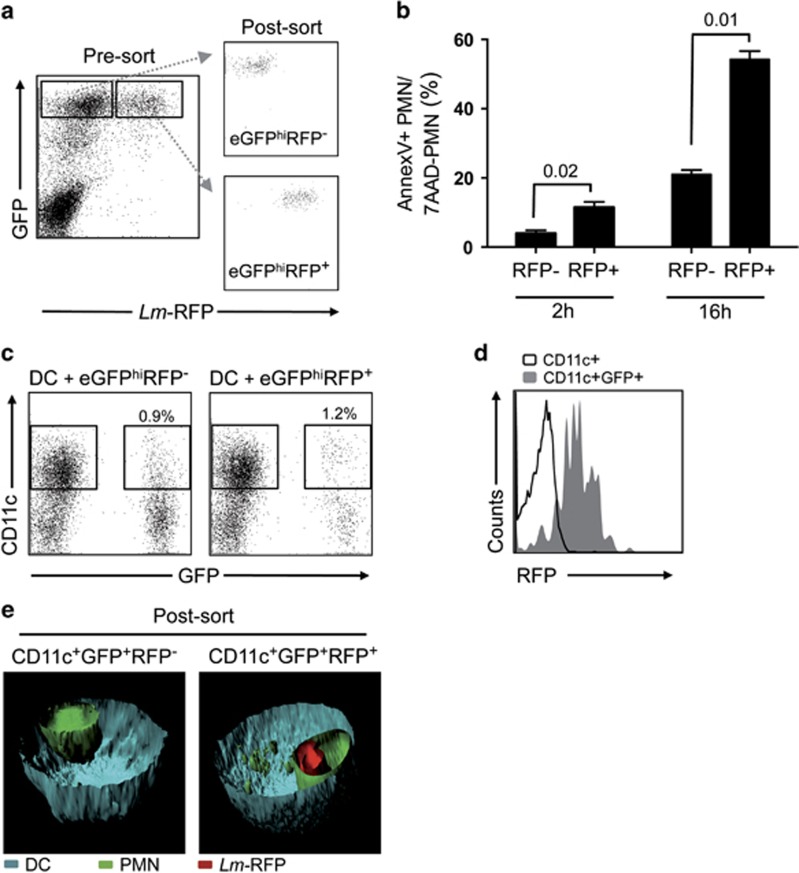
Uptake of infected and uninfected neutrophils by DCs. (**a**) The eGFP^hi^ neutrophils recovered from the ear dermis 12 h after infection of C57BL/6 LYS-eGFP mice with 2 × 10^6^ *Lm*-RFP metacyclic promastigotes were sorted to obtain uninfected eGFP^hi^RFP^**−**^ and infected eGFP^hi^RFP^**+**^ neutrophils. Data are representative of several independent experiments. (**b**) Frequencies of annexinV^**+**^ 7-AAD^**−**^ uninfected eGFP^hi^RFP^**−**^ or infected eGFP^hi^RFP^**+**^ neutrophils recovered from the ear dermis at 2 and 16 h after infection with 2 × 10^6^ *Lm*-RFP metacyclic promastigotes. Values shown are the mean percentage±1 S.D., *n*=3–4 ears/group/time point. Data are representative of two independent experiments. (**c**) Representative dot plots of DCs cultured with eGFP^hi^RFP^**−**^ and eGFP^hi^RFP^**+**^ dermal neutrophils for 16 h (1 : 2 neutrophil/DC ratio) and analyzed for expression of CD11c and GFP signal. Gates represent populations of CD11c^+^GFP^−^ and CD11c^+^GFP^+^ cells. (**d**) Representative histogram plots of RFP expression by post-sorted populations of CD11c^+^GFP^-^ (black line) and CD11c^+^GFP^+^ (gray filled) cells recovered from co-culture of DCs with eGFP^hi^RFP^**+**^ dermal neutrophils, gated as shown in (**c**). (**e**) Confocal microscopic images of sorted CD11c^+^GFP^+^ cells recovered from culture of DCs with eGFP^hi^RFP^**−**^ and eGFP^hi^RFP^**+**^ dermal neutrophils. DCs were derived from *β*-actin CFP mice and are shown in blue, eGFP^hi^ neutrophils are shown in green and *Lm*-RFP parasites are shown in red

To study the interaction and uptake of neutrophils by DCs *in vitro*, metacyclic promastigotes prepared from a red fluorescent protein (RFP)-expressing strain of *L. major* (*Lm*-RFP) were injected into the ear dermis of LYS-eGFP mice.^13^ The recovery of homogeneous populations of infected and uninfected neutrophils from the skin by sorting the eGFP^hi^RFP^+^ and eGFP^hi^RFP^−^ cells (Figure 1a), and the accelerated apoptosis of the *L. major*-infected neutrophils, have been previously described.^10, 11^ Consistently, Annexin V staining of neutrophils recovered from the skin at 2 and 16 h after infection confirmed the accelerated exposure of phosphatidylserine (PtdSer) on infected neutrophils (Figure 1b). Comparison of Annexin V-stained cells before and after sorting revealed a slightly greater proportion of neutrophils acquired this marker during the sorting procedure, but in each case the proportion of infected neutrophils that stained positive was significantly greater than the uninfected cells (Supplementary Figure S1).

The infected and uninfected neutrophils recovered 12 h after infection (Figure 1a) were cultured with bone marrow-derived dendritic cells, hereafter called DCs. After 16 h of co-culture, a minor population of CD11c^+^GFP^+^ cells was detected in each case (Figure 1c), suggesting that dermal-derived *L. major-*infected and uninfected neutrophils were captured by DCs *in vitro*. The population of CD11c^+^GFP^+^ cells represents 3–14% of the CD11c^+^ cells, depending on the experiment (Supplementary Figure S2). Of the CD11c^+^GFP^+^ cells originating from the co-culture of DCs with eGFP^hi^RFP^+^-infected neutrophils, the majority were RFP^+^, indicating that the infected neutrophils were engulfed by the DCs before their release or killing of their internalized parasites (Figure 1d). In order to visualize the CD11c^+^GFP^+^ cells by confocal microscopy, we employed bone marrow-derived DCs from *β*-actin-CFP mice. After sorting and 3D reconstruction from a stack of serial cross-sections of the cells, we could confirm that at least some of the DCs had internalized the eGFP^hi^RFP^+^-infected neutrophils and eGFP^hi^RFP^−^-uninfected neutrophils (Figure 1e).

### *L. major*-infected neutrophils inhibit DC maturation and their subsequent function as APCs

**Figure 2:**
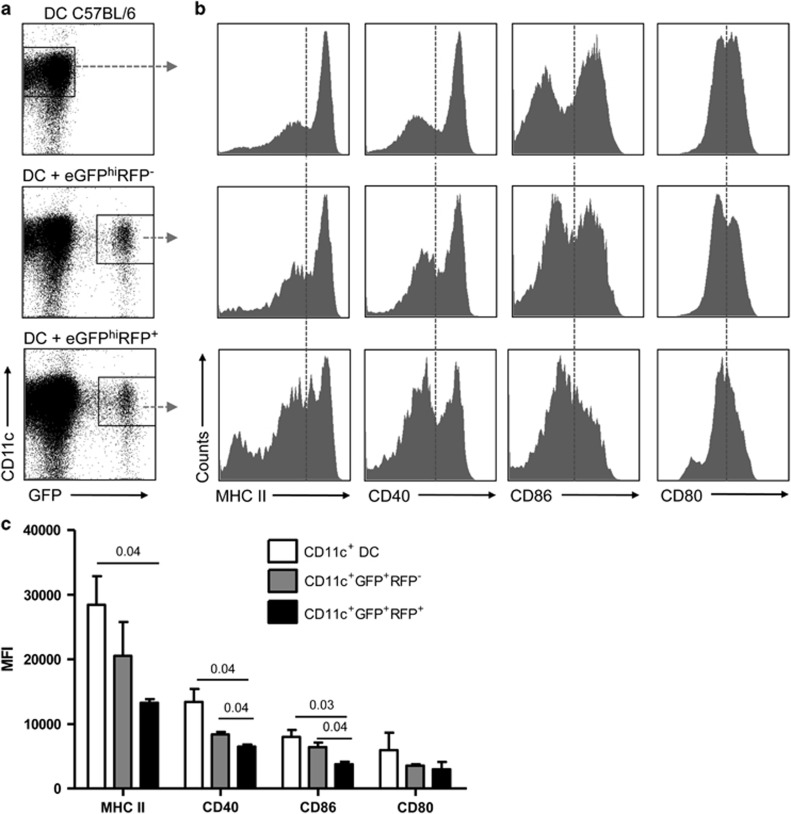
Activation markers on DCs following capture of infected and uninfected neutrophils. (**a**) Representative dot plots of DCs cultured or not with eGFP^hi^RFP^**−**^ or eGFP^hi^RFP^**+**^ dermal neutrophils for 12 h and analyzed for expression of CD11c and GFP signal. Gates represent populations of CD11c^+^ and CD11c^+^GFP^+^ cells. (**b**) Representative histogram plots of CD11c^+^ and CD11c^+^GFP^+^ gated cells stained for MHC II, CD40, CD86 and CD80. Data are representative of three independent experiments. (**c**) Mean fluorescence intensity (MFI) of MHC II, CD40, CD86 and CD80 expression on CD11c^+^ DC (white bars), CD11c^+^GFP^+^RFP^−^ (gray bars) and CD11c^+^GFP^+^RFP^+^ (black bars) gated cells. Mean MFI±1 S.D. calculated from three independent experiments

**Figure 3:**
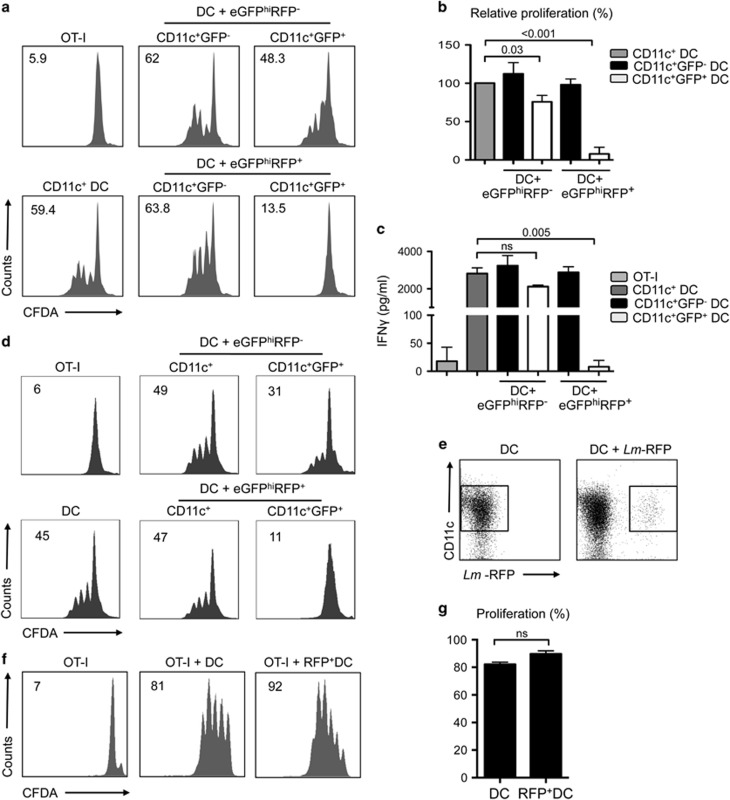
Antigen presentation function of DCs that have captured infected and uninfected neutrophils. (**a–c**) Sorted CD11c^+^ (no exposure to neutrophils), CD11c^+^GFP^−^ and CD11c^+^GFP^+^ cells (following overnight culture with uninfected or infected neutrophils) were cultured with CFDA-labeled OT-I CD8^+^ T cells for 3 days in the presence of soluble OVA. (**a**) Representative histogram plots of CFDA fluorescence of CD8^+^CD3^+^ gated cells showing the proliferative response. Numbers represent the frequency of cells with reduced CFDA content. (**b**) CFDA-labeled OT-I CD8^+^ T cells with reduced CFDA content (mean percentage±1 S.E.M., calculated from the means of three independent experiments, two replicates per experiment). (**c**) IFN-*γ* levels in supernatants of the cultures described in (**b**) (mean concentration±1 S.E.M., calculated from the means of three independent experiments, two replicates per experiment). (**d**) Histogram plots of CFDA fluorescence of OT-I CD8^+^CD3^+^ gated cells showing the proliferative response following 3 days of co-culture with CD11c^+^, CD11c^+^GFP^−^ and CD11c^+^GFP^+^ cells in the presence of SIINFEKL peptide. Numbers represent the frequency of cells with reduced CFDA content. (**e**) Representative dot plots showing the gates used for sorting of the CD11c^+^ (no exposure to *Lm*-RFP) and CD11c^+^RFP^+^ cells following direct infection with *Lm*-RFP metacyclic promastigotes for 12 h (1 : 1 parasite/DC ratio). (**f**) Representative histogram plots of gated CFDA-labeled OT-I CD8^+^ T cells showing the percentage of the proliferative response after co-culture with CD11c^+^ and infected CD11c^+^RFP^+^ cells for 3 days in the presence of OVA antigen. (**g**) CFDA-labeled OT-I CD8^+^ T cells with reduced CFDA content (mean percentage± 1 S.D., calculated from three independent experiments)

To evaluate whether the uptake of dermal eGFP^hi^RFP^−^-uninfected or eGFP^hi^RFP^+^-infected neutrophils by DCs would result in modulation of DC function, we cultured DCs with purified dermal eGFP^hi^RFP^−^-uninfected and eGFP^hi^RFP^+^-infected neutrophils in a 2 : 1 DC/neutrophil ratio overnight in complete RPMI with 10% FCS (Figure 2a) and determined the expression level of DC maturation markers. Uptake of eGFP^hi^RFP^+^-infected neutrophils by DCs reduced their expression of MHC class II, CD40 and CD86 compared with control DCs and, surprisingly, also the expression of CD40 and CD86 in comparison with DCs that had captured eGFP^hi^RFP^−^-uninfected neutrophils (Figures 2b and c). Expression of CD80 was not affected. To investigate their antigen-presentation capacity, an additional sorting was performed yielding four different populations of DCs: CD11c^+^GFP^−^ cells that had not taken up neutrophils during their overnight culture with either eGFP^hi^RFP^−^-uninfected or eGFP^hi^RFP^+^-infected neutrophils, and CD11c^+^GFP^+^-positive cells that had taken up either uninfected or infected neutrophils. CD11c^+^ cells that had not been cultured with neutrophils were submitted to the same sorting procedure as controls. The DCs were cultured with naive CFDA-labeled OT-I CD8^+^ T cells and soluble ovalbumin (OVA) for 3 days (Figures 3a and b). The control CD11c^+^ DCs induced strong proliferation, with 59% of the OT-I cells showing dilutions in CFDA content. Similar levels of OT-I proliferation (62 and 63.8%) were induced by the CD11c^+^GFP^−^ DCs recovered from the neutrophil co-cultures. The CD11c^+^GFP^+^ DCs that had captured the eGFP^hi^RFP^−^-uninfected neutrophils showed a slight reduction in the OT-I proliferation induced (48.3%). In contrast, the DCs that had taken up the eGFP^hi^RFP^+^-infected neutrophils were strongly inhibited in their capacity to stimulate OT-I proliferation (13.5%) and IFN-*γ* production (Figures 3a–c). Complete suppression of OT-I priming was still observed when the DCs that had taken up infected neutrophils were pulsed with SIINFEKL peptide (Figure 3d), indicating that the dysfunction was not confined to the generation of the immunogenic peptide. Blocking the apoptotic process of purified dermal eGFP^hi^RFP^+^-infected neutrophils by fixing the cells with 2% paraformaldehyde before their co-culture with DCs partially reversed their ability to inhibit DC antigen presentation function (Supplementary Figure S3). Importantly, sort-purified infected DCs that had directly taken up *L. major*-RFP metacyclic promastigotes efficiently presented soluble OVA to CFDA-labeled OT-I CD8^+^ T cells (92%), comparable to the proliferative response observed using uninfected DCs (Figures 3e–g). These findings suggest that the inability of DCs to function as APCs following their capture of infected neutrophils is not due to their parasitization *per se*.

**Figure 4:**
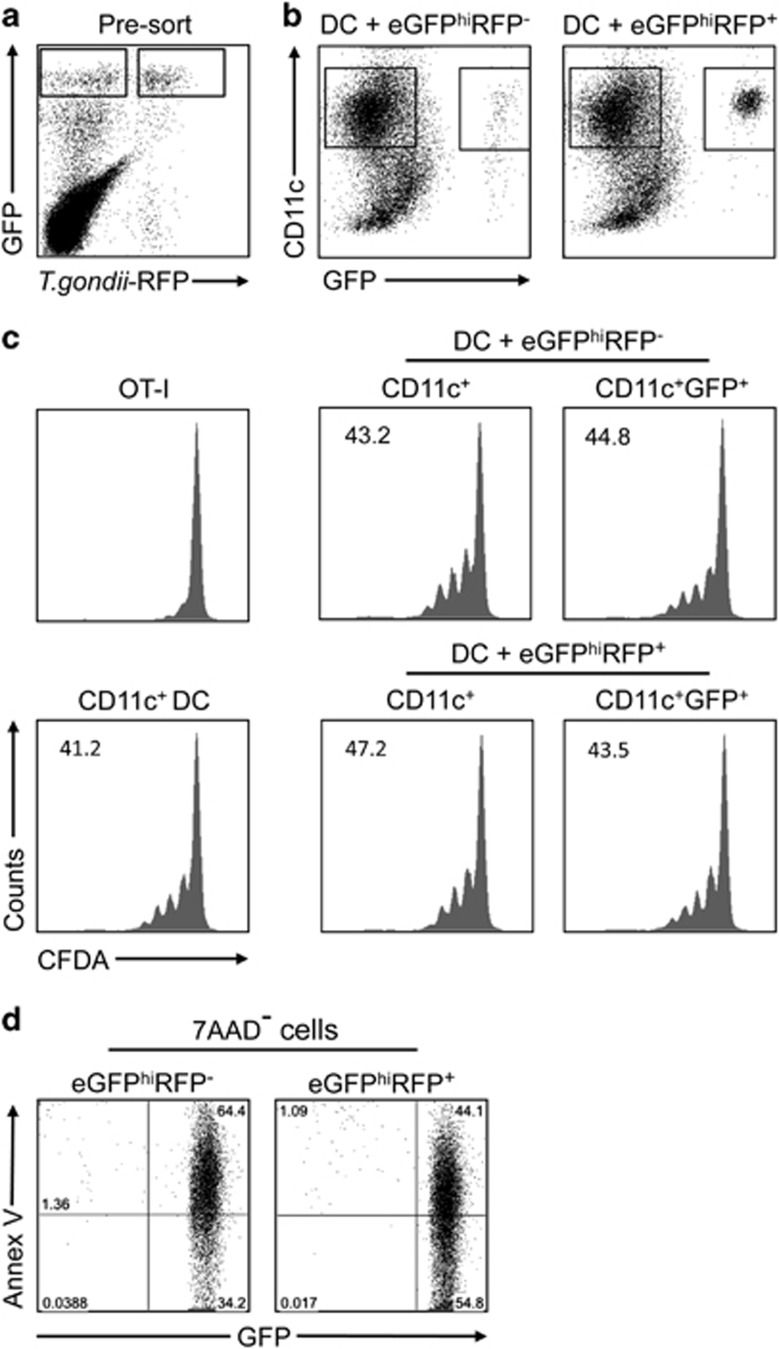
Antigen presentation function of DCs that have captured *T. gondii*-infected and uninfected neutrophils. (**a**) eGFP^hi^RFP^**−**^-uninfected and eGFP^hi^RFP^**+**^-infected neutrophils recovered from the ear dermis 12 h after infection with 1 × 10^6^ *T. gondii*-RFP. (**b**) Representative dot plots of DCs cultured or not with eGFP^hi^RFP^**−**^-uninfected and eGFP^hi^RFP^**+**^ *T. gondii*-infected dermal neutrophils for 12 h and analyzed for expression of CD11c and GFP signal. Gates represent populations of CD11c^+^GFP^−^ and CD11c^+^GFP^+^ cells. (**c**) Sorted CD11c^+^, CD11c^+^GFP^−^ and CD11c^+^GFP^+^ cells were cultured with CFDA-labeled OT-I CD8^+^ T cells for 3 days in the presence of OVA antigen. Representative histogram plots of CFDA fluorescence of CD8^+^CD3^+^ gated cells showing the proliferative response. Numbers represent the frequency of cells with reduced CFDA content. (**d**) Representative dot plots of sorted eGFP^hi^RFP^**−**^-uninfected and eGFP^hi^RFP^**+**^-infected neutrophils recovered from the ear dermis 12 h after infection with 1 × 10^6^ *T. gondii*-RFP parasites and stained with annexin V-APC after gating on 7-AAD^**−**^ cells. Quadrant values show the percentage of total gated cells. The data are representative of two independent experiments

To address whether the antigen presentation function of DCs is also compromised after capture of inflammatory neutrophils infected by a different organism, a RFP-expressing strain of *Toxoplasma gondii* was injected into the ears of LYS-eGFP mice. After 12 h, eGFP^hi^RFP^+^-infected and eGFP^hi^RFP^−^-uninfected neutrophils (Figure 4a) were sorted and cultured with DCs overnight (Figure 4b). Following an additional sort to obtain populations of DCs that had captured or not infected or uninfected neutrophils, the DCs were cultured with naive CFDA-labeled OT-I CD8^+^ T cells in presence of soluble OVA. DCs that had not been co-cultured with neutrophils but submitted to the sorting procedure were again used as controls. All of the DCs induced strong and comparable levels of OT-I CD8^+^ T-cell proliferation, including the DCs that had taken up eGFP^hi^RFP^+^ *T. gondii*-infected neutrophils (Figure 4c). Interestingly, the expression of PtdSer on neutrophils recruited to the ear dermis by *T. gondii* inoculation was detected at relatively high frequency on both infected and uninfected cells (Figure 4d), consistent with a prior report showing high levels of apoptosis elicited by *T. gondii* through overproduction of proinflammatory cytokines.^14^

**Figure 5:**
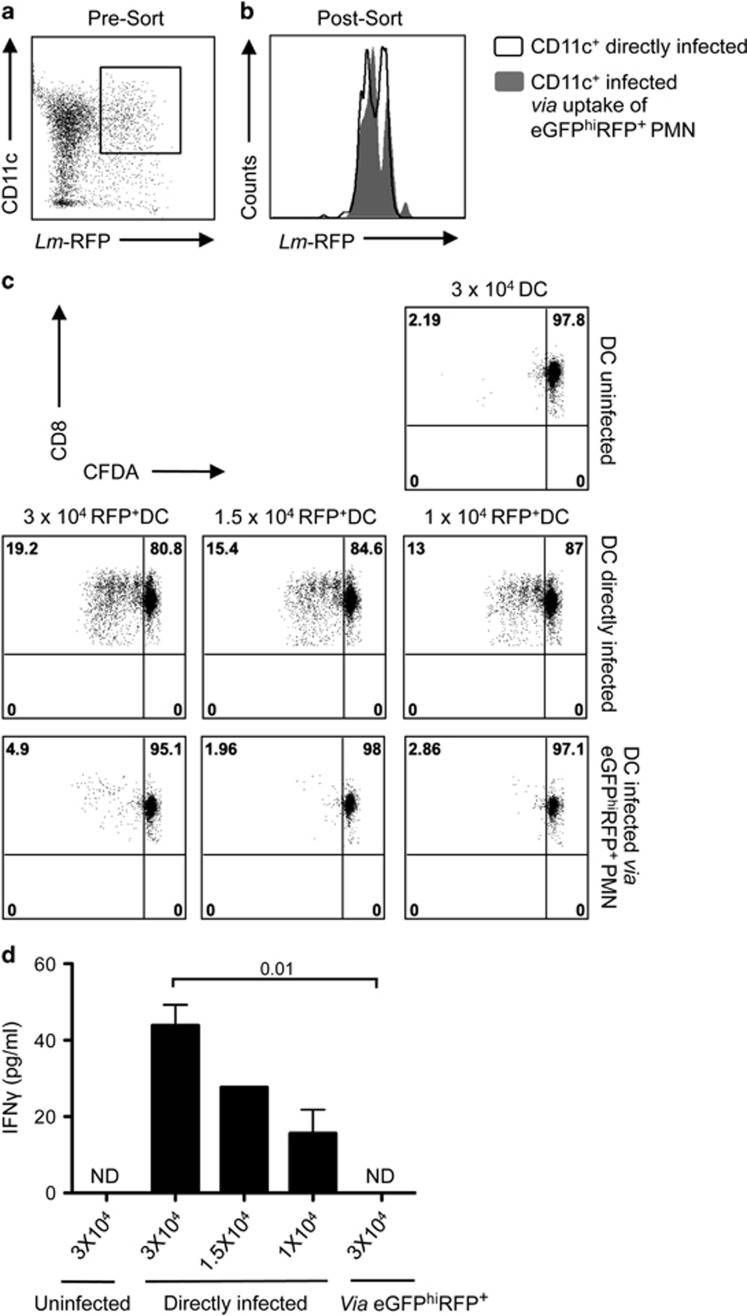
Presentation of parasite-derived antigen by DCs that have captured *L. major*-infected neutrophils. (**a–d**) DCs were infected for 12 h with *Lm*-NT-OVA-RFP directly (1 : 1 parasite/DC ratio) or via uptake of eGFP^hi^RFP^+^-infected (*Lm*-NT-OVA-RFP) neutrophils (1 : 1 neutrophil/DC ratio). (**a**) Representative dot plot showing the gate used for sorting of CD11c^+^RFP^+^-infected cells. (**b**) Representative histogram plots of RFP expression by CD11c^+^ cells directly infected with *Lm*-NT-OVA-RFP (black line) and CD11c^+^ cells infected via uptake of eGFP^hi^RFP^+^-infected neutrophils (gray filled). (**c**) Representative dot plots from one of two independent experiments of CFDA fluorescence of OT-I CD8^+^ T-cell gated populations 3 days after culture with different numbers of uninfected or directly infected DCs or DCs infected via uptake of eGFP^hi^RFP^+^ neutrophils. Numbers represent the frequency of cells in each quadrant. (**d**) IFN-*γ* levels in supernatant of the cultures described in (**c**) (mean concentration±1 S.D.) The data are representative of two independent experiments

We next investigated the ability of DCs that had captured infected neutrophils to present parasite-derived antigen. eGFP^hi^RFP^+^-infected neutrophils were sorted 12 h after intradermal (i.d.) injection of LYS-eGFP mice with a *Lm*-RFP line engineered to secrete a chimeric OVA protein, inclusive of the SIINFEKL OT-I epitope (*Lm*-NT-OVA-RFP). Following overnight culture, the RFP^+^DCs infected via uptake of eGFP^hi^RFP^+^-parasitized neutrophils (1 : 1 neutrophil/DC ratio) or infected directly with *Lm*-NT-OVA-RFP (1 : 1 parasite/DC ratio) were sorted based on CD11c expression and RFP signal, yielding two populations of infected DCs that were comparable in their RFP signals (Figures 5a and b). Following co-culture with CFDA-labeled OT-I CD8^+^ T cells, the DCs that were directly infected with *Lm*-NT-OVA-RFP stimulated low but significantly elevated levels of proliferation and IFN-*γ* secretion compared with uninfected DCs at all DC/OT-I ratios used (Figures 5c and d). The DCs infected via uptake of parasitized neutrophils induced a slight proliferative response only at the highest DC/OT-I ratio used, and no IFN-*γ* secretion in any of the cultures (Figures 5c and d).

### Inhibition of APC function following capture of *L. major*-infected neutrophils requires signaling via the receptor tyrosine kinase Mer

**Figure 6:**
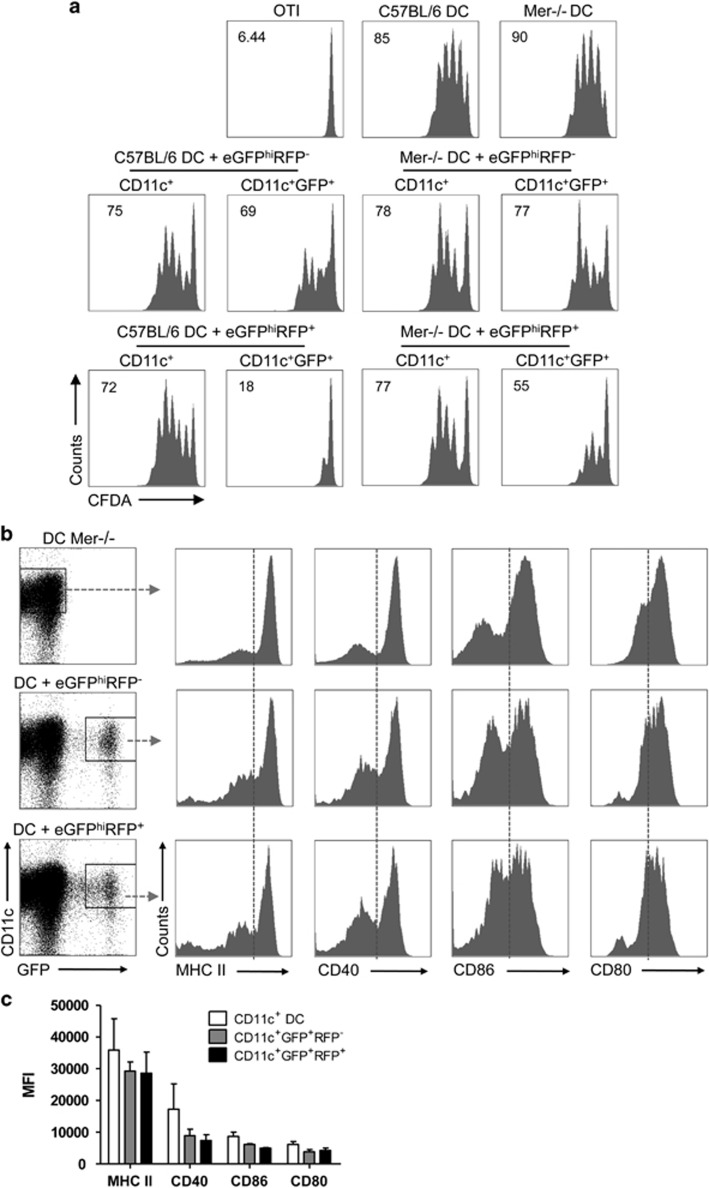
Antigen presentation function of Mer-deficient DCs following capture of *L. major*-infected and uninfected neutrophils. C57BL/6 and Mer ^−/−^ DCs were cultured or not with eGFP^hi^RFP^−^-uninfected and eGFP^hi^RFP^+^-infected dermal neutrophils for 12 h. Sorted CD11c^+^, CD11c^+^GFP^−^ and CD11c^+^GFP^+^ cells were cultured with CFDA-labeled OT-I CD8^+^ T cells for 3 days in the presence of OVA antigen. (**a**) Representative histogram plots of CFDA fluorescence of CD8^+^CD3^+^ gated cells showing the proliferative response. Numbers represent the frequency of cells with reduced CFDA content. (**b**) Representative dot plots of Mer^−/−^ DCs cultured or not with eGFP^hi^RFP^-^ and eGFP^hi^RFP^**+**^ dermal neutrophils for 12 h and analyzed for expression of CD11c and GFP signal. Representative histogram plots of the respective gated populations of CD11c^+^ and CD11c^+^GFP^+^ cells, and stained for MHC II, CD40, CD86 and CD80, are shown. The data are representative of three independent experiments. (**c**) Mean fluorescence intensity (MFI) of MHC II, CD40, CD86 and CD80 expression on CD11c^+^ DC (white bars), CD11c^+^GFP^+^RFP^−^ (gray bars) and CD11c^+^GFP^+^RFP^+^ (black bars) gated cells. Mean MFI±1 S.D. calculated from three independent experiments

The *in vitro* and *in vivo* studies have suggested that the receptor tyrosine kinase Mer plays a critical role in mediating apoptotic cell-induced inhibition of DC activation/maturation.^15, 16^ Mer was uniformly detected on bone marrow-derived DCs and dermal DCs (Supplementary Figure S4). To investigate whether the modulation of DC function following uptake of infected neutrophils is associated with Mer receptor signaling, Mer^−/−^ or C57BL/6 (wt) mice were used as a source of bone marrow-derived DCs. The capture of infected neutrophils by wt DCs was again associated with virtually complete inhibition of their ability to stimulate OT-I proliferation in response to soluble OVA (Figure 6a). In contrast, Mer^−/−^ DCs that had captured infected neutrophils retained most of their capacity to stimulate OT-I proliferation. The ability of Mer^−/−^ DCs harboring infected neutrophils to retain their cross-presentation capacity was associated with unaltered expression of MHC class II, CD40 and CD80 compared with control DCs or DCs that had taken up uninfected neutrophils; CD86 expression was only slightly reduced (Figures 6b and c).

We explored the role of another receptor, CD300f, also involved in apoptotic cell clearance and in negative regulation of DC activation,^17, 18^ to determine whether engagement of this receptor might also be required to suppress the cross-presentation function of DCs following their uptake of infected neutrophils *in vitro*. Employing DCs prepared from CD300f^−/−^ mice, no recovery of their ability to induce OT-I proliferation was observed (Supplementary Figure S5). No difference was observed in the frequency of neutrophil uptake by either Mer^−/−^ DCs or CD300f^−/−^ DCs compared with wild-type DCs during the 12 h co-culture (data not shown), reflecting the redundancy of receptors involved in this process.^19^

**Figure 7:**
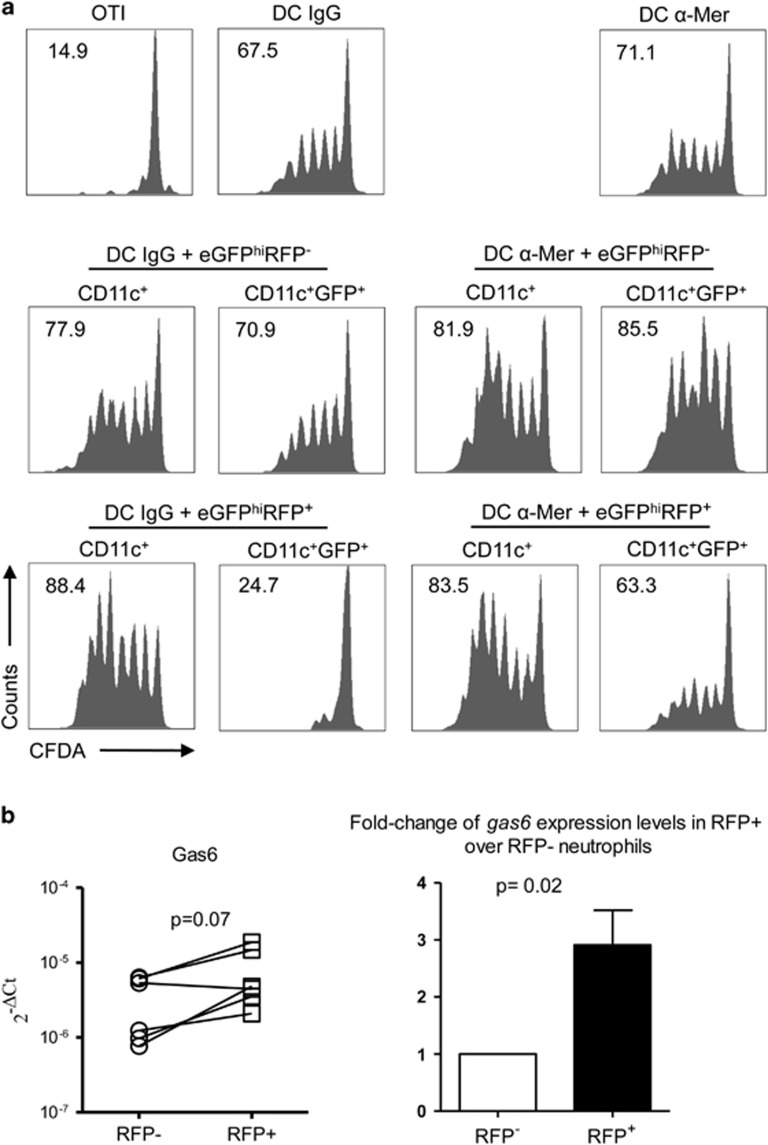
Antibody blockade of Mer rescues the antigen presentation function of DCs. (**a**) C57BL/6 DCs were pretreated with isotype control or anti-Mer antibody for 1 h, then cultured or not with eGFP^hi^RFP^−^ and eGFP^hi^RFP^+^ dermal neutrophils for 12–16 h. Sorted CD11c^+^, CD11c^+^GFP^−^ and CD11c^+^GFP^+^ cells were cultured with CFDA-labeled OT-I CD8^+^ T cells for 3 days in the presence of OVA antigen. Representative histogram plots of CFDA fluorescence of CD8^+^CD3^+^ gated cells showing the proliferative response. Numbers represent the frequency of cells with reduced CFDA content. The data are representative of two independent experiments. (**b**) Gas6 mRNA expression in uninfected eGFP^hi^RFP^**−**^ and *L. major-*infected eGFP^hi^RFP^**+**^ neutrophils purified from a total of 6 independent sorting procedures, each combining dermal cells pooled from 10 ears, and shown as relative expression levels in paired samples or as fold change in relative expression levels (mean±1 S.D.)

To further implicate Mer signaling in the inhibition of APC function observed, we treated C57BL/6 DCs with blocking anti-Mer or isotype control antibodies before and during co-culture with infected or uninfected neutrophils. Again, only the DCs that had captured infected neutrophils, but not uninfected neutrophils, were impaired in their ability to cross-present soluble OVA (Figure 7a). The response was substantially rescued by treatment with the anti-Mer antibody.

The known ligands for Mer and other TAM receptors are growth-arrest-specific protein 6 (Gas6) and protein S, large (80 kDa) soluble proteins with high homology that serve as bridging molecules linking a TAM receptor to exposed PtdSer on the apoptotic cell.^20, 21^ We were unable to stain for either of these ligands on neutrophils recovered from the ear dermis, although their presence may have been below the detection limits of the assay, in so far as the surface staining of these proteins on apoptotic cells has only rarely been reported.^16^ We were, however, able to detect differences in the level of Gas6 mRNA expression that was greater in infected compared with uninfected neutrophils sorted from the same inoculation site in 5 of 6 independent experiments and averaged a significant threefold increase (*P*=0.02; Figure 7b).

We also evaluated whether *L. major*-infected and uninfected neutrophils release different soluble factors that may affect DC function. The *ex vivo* analysis by RT-PCR of different cytokines, including IL-10, IL-6, TGF*β* and IL-12p40, revealed a 10-fold increase in mRNA for IL-10 in sorted eGFP^hi^RFP^+^-infected neutrophils compared with eGFP^hi^RFP^−^-uninfected neutrophils and eGFP^hi^ neutrophils recruited after dermal injection of LPS (Supplementary Figure S6A). Intracellular staining for cytokines in neutrophils recovered 12 h after *Lm*-RFP injection revealed only low frequencies of positive cells, except for IL-6 for which a high proportion of positive cells were present within both the infected and uninfected neutrophil populations (Supplementary Figure S6B). IL-10 reporter mice were used to facilitate more sensitive detection of neutrophil IL-10 expression, but still only low frequencies of positive cells were observed (Supplementary Figure S6C).

## Discussion

In inflammatory settings, such as those associated with infection or tissue injury, the active clearance of dying cells, mainly neutrophils, by a phagocytic process termed efferocytosis is central to the resolution of acute inflammation and to tissue repair (reviewed in Bratton and Henson^22^). The clearance of dying cells has only rarely been studied in the context of infection transmitted by the bite of arthropod vectors that will in most instances provoke a massive inflammatory, wound healing response in the skin. Following inoculation of *L. major* by sand fly bite or by needle, neutrophils are the predominant recruited and infected cells in the early stages of infection in the skin, and depletion of neutrophils hastens the development of Th1-mediated immunity.^10, 11, 12, 23^ A recent paper employing neutropenic *Genista* mice also reported enhanced Th1 responses and immunity, in this case to infection with a New World cutaneous strain of *Leishmania mexicana*.^24^ Thus, the acute neutrophilic response appears to be exploited by *Leishmania* to delay the onset of acquired resistance. We have presented *in vivo* evidence to suggest that the majority of infected DCs recovered from the skin immediately after *L. major* challenge acquire their infections via capture of infected neutrophils, and that these encounters are responsible for inhibiting DC activation and their ability to initiate the anti-*Leishmania* T-cell response.^11^ In the current studies, we model the interaction of skin-recovered, infected or noninfected neutrophils with bone marrow-derived DCs *in vitro* to directly assess the ability of DCs to capture these cells. We further address the consequences of neutrophil capture by DCs on their ability to cross-present exogenous or parasite-derived antigen to CD8^+^ T cells *in vitro*. The role of cross-presenting DCs in protection against *L. major* has been recently described.^25^ Our *in vitro* studies reveal profound impairment of T-cell priming following capture of infected but not uninfected neutrophils that is dependent on engagement of the tyrosine kinase receptor Mer on the DCs.

Our data draw a clear distinction between the ability of infected and uninfected neutrophils recovered from the same *L. major*-loaded dermis to deliver inhibitory signals to DCs. The engulfment of uninfected neutrophils was not a sufficient condition to inhibit DC maturation and cross-presentation, whereas the uptake-infected neutrophils was powerfully suppressive. The difference could not be attributed to any direct effect that the parasite has on the DCs, as DCs that had taken up *Lm-*RFP promastigotes directly were fully functional for cross-presentation of exogenous OVA, and for OVA secreted by *Lm-*RFP. It appears that the infected and noninfected neutrophils differ in the manner in which their cell deaths are evaluated by the DCs. At the time of their recovery from the inoculated dermis, the infected neutrophils displayed a higher frequency of Annexin V and TUNEL staining cells (Figure 1),^11^ indicating an accelerated apoptotic program. The phagocytosis of certain bacteria by neutrophils is also known to accelerate their apoptosis at sites of infection (reviewed in DeLeo^26^). Phagocytosis-induced neutrophil apoptosis is dependent on the activation of effector caspases initiated by death receptor signals received during microbial attachment and uptake,^27^ whereas the uninfected neutrophils may die by a spontaneous apoptotic program initiated by their culture in the absence of prosurvival factors. Nonetheless, it is clear that phagocytosis *per se* is not a sufficient condition to endow infected, dying neutrophils with the ability to engage inhibitory receptors on DCs, as *L. major-*infected neutrophils that were fixed before their capture by DCs were less inhibitory, and skin-recovered neutrophils harboring *T. gondii* were not inhibitory. Furthermore, in some infection-driven, inflammatory settings, including *Candida albicans*, *Salmonella typhimurium* and *M. tuberculosis*, the clearance of dying, infected cells can contribute immunogenic signals to the antimicrobial response.^28, 29, 30, 31, 32^ The outcome with regard to the immune response may vary depending on whether maturation signals are also received (reviewed in Green *et al.*^33^).

Accumulating evidence points to a critical role for a subfamily of receptor tyrosine kinases, Tyro3, Axl and Mer (TAM), in promoting the clearance of apoptotic cells and in negatively regulating innate immune functions (reviewed in Lemke and Burstyn-Cohen^34^). Mice lacking all three TAM receptors develop a massive lymphoproliferative disorder and systemic autoimmunity. Mer in particular is used by DCs to transduce inhibitory signals in response to encounters with apoptotic cells, resulting in suppression of proinflammatory cytokine production, DC maturation and antigen cross-presentation function.^15, 16^ We could detect Mer expression on the majority of bone marrow-derived DCs as well as DCs recovered from the ear dermis. More importantly, we could substantially reconstitute the cross-priming capacity of DCs that had engulfed infected neutrophils by using antibodies to block engagement of Mer, or by using bone marrow-derived DCs prepared from Mer^−/−^ mice. In neither case was there a defect in neutrophil uptake by the DCs, consistent with other studies showing that DCs do not require Mer for the phagocytosis of apoptotic cells,^15, 35^ and with the fact that multiple and redundant receptors for PtdSer and other ‘eat me' signals are known to be constitutively expressed or induced on phagocytes, including T-cell Ig mucins (TIM1 and TIM4), BAI1, MFGGE8 and CD300 family members (reviewed in Ravichandran and Lorenz^19^). The TAM receptors are activated by two closely related ligands, Gas6 and protein S, each containing glutamic acid-rich domains that bind to PtdSer on the apoptotic cell.^20, 21^ Although we could find no evidence for differential display of either of these ligands on the surface of the infected and uninfected neutrophils, Gas6 mRNA was significantly elevated in the infected cells, suggesting that the infected neutrophils might upregulate production of Gas6 that binds to the cell in an autocrine manner, but below the sensitivity of detection by surface staining. The enzymatic digestion of the ear tissue required to obtain the dermal cells may have also compromised surface detection of Gas6. We explored the role of another PtdSer-recognition receptor, CD300f, that also promotes phagocytosis of apoptotic cells, contains tyrosine-based signaling motifs in its cytoplasmic tail and has been shown to negatively regulate DC function.^17, 18^ In contrast to the DCs lacking Mer, DCs from CD300f^−/−^ mice that had captured infected neutrophils were still strongly suppressed in their ability to cross-present OVA to OT-I cells.

The function of Mer has been recently refined as a tolerogenic receptor specialized for the clearance of apoptotic cells in homeostatic as opposed to inflammatory environments.^36^ In this context, the uptake of *L. major* by inflammatory neutrophils in the skin may reset their apoptotic program to resemble homeostatic cell death. In contrast, inflammatory neutrophils that remain noninfected, or that are infected by certain other microbes, for example, *T. gondii*, die in a manner that is evaluated by the DC as immunogenic, or at least nontolerogenic, permitting the host to mount an adaptive, antimicrobial response. By confining their early encounters with DCs to the clearance of infected, apoptotic neutrophils, our findings provide a clear set of cellular interactions that explain how *L. major* delays the development of acquired resistance to establish infection and promote disease.

## Materials and Methods

### Mice

This study was carried out in strict accordance with the recommendations in the Guide for the Care and Use of Laboratory Animals of the National Institutes of Health. The protocol was approved by the Animal Care and Use Committee of the NIAID, NIH (protocol number LPD 68E). All mice were maintained at the NIAID animal care facility under specific pathogen-free conditions. Female C57BL/6 and C57BL/6 RAG1-deficient OT-I CD8^+^ TCR transgenic mice were purchased from Taconic Laboratories (Hudson, NY, USA). *β*-Actin-CFP Tg(ACTB-ECFP)1 Nagy/J mice were purchased from the Jackson Laboratory (Bar Harbor, ME, USA). C57BL/6 LYS-eGFP knock-in mice^13^ were bred at Taconic Laboratories. Mer^−/−^, CD300f^−/−^ and IL-10/eGFP mice, all on the C57BL/6 background, were gifts from C Rothlin (Yale University, New Haven, CT, USA), J Coligan (NIAID, Bethesda, MD, USA) and C Karp (Cincinnati Children's Hospital, Cincinnati, OH, USA) respectively, and were bred in the NIAID animal breeding facility.

### Parasites

*L. major* infections were carried out using different transfected lines of *L. major* Friedlin strain FV1 (MHOM/IL/80/FN): a stable transfected line of *L. major* FV1 promastigotes expressing a red fluorescent protein (*Lm*-RFP) generated as described previously,^37^ and *Lm*-RFP expressing a portion of the ovalbumin gene encoding amino acids 232 to 288 containing the MHC class I-restricted OVA257-264 (SIINFEKL) epitope generated by transfection of the *Lm*-RFP line with the pKS NEO NT-OVA plasmid (*Lm*-NT-OVA-RFP), as described previously.^37, 38^ For *T. gondii* infections, parental ME-49 (clone C1) was transfected with RFP using a modified plasmid construct as previously described,^39^ and kindly provided by M Grigg (NIAID, Bethesda, MD, USA).

### Parasites preparation and intradermal inoculation

*L. major*-transfected lines were grown at 26 °C in medium 199 supplemented with 20% heat-inactivated FCS (Gemini Bio-Products, Woodland, CA, USA), 100 U/ml penicillin, 100 *μ*g/ml streptomycin, 2 mM L-glutamine, 40 mM Hepes, 0.1 mM adenine (in 50 mM Hepes), 5 mg/ml hemin (in 50% triethanolamine), 1 mg/ml 6-biotin (M199/S) and 50 *μ*g/ml of Geneticin (G418, Gibco, Woodland, CA, USA). The *Lm*-NT-OVA-RFP was grown in culture medium supplemented with hygromycin B (100 *μ*g/ml) and Geneticin (25 *μ*g/ml). Infective-stage metacyclic promastigotes of *L. major* were isolated from stationary cultures (4–6 days old) by negative selection of noninfective forms using peanut agglutinin (PNA, Vector Laboratories Inc., Burlingame, CA, USA). *T. gondii*-RFP were routinely passaged *in vitro* in monolayers of human foreskin fibroblasts (HFFs) at 37 °C in the presence of 5% CO_2_, spun and washed before experiments. Mice were injected with the specified number of parasites in the ear dermis by i.d. injection using a 29 1/2 GA needle in a volume of 10 *μ*l.

### Processing of ear tissue, neutrophil isolation and staining

Ear tissue was prepared as previously described.^40^ Briefly, the two sheets of infected ear dermis were separated, deposited in DMEM containing 100 U/ml penicillin, 100 *μ*g/ml streptomycin and 0.2 mg/ml Liberase CI purified enzyme blend (Roche Diagnostics Corp., Chicago, IL, USA) and incubated for 1 h and 30 min at 37 °C. Digested tissue was placed in a grinder and processed in a tissue homogenizer (Medimachine; Becton Dickenson, Chicago, IL, USA). To obtain neutrophils recruited to the site of infection in the skin, LYS-eGFP mice were inoculated in the ear dermis with 1–2 × 10^6^ *Lm-*RFP or *Lm*-NT-OVA-RFP or 1 × 10^6^ *T. gondii*-RFP parasites in 10 *μ*l. After 12 h, ear tissue was prepared as described above and infected (eGFP^hi^RFP^+^) and uninfected (eGFP^hi^RFP^−^) neutrophils were sorted from dermal tissue using a FACSVantage or a FACsAria (BD Biosciences, Chicago, IL, USA) cell sorter. Sorted populations were washed once and immediately analyzed for apoptotic markers or cultured with mouse BM-DCs. Presorted neutrophil populations recruited to the site at 2 and 16 h after infection were stained with Annexin-V-APC and 7-AAD (BD Biosciences), and cells were analyzed by flow cytometry.

### Generation of bone marrow-derived DCs and co-culture with neutrophils

C57BL/6, *β*-actin-CFP, Mer^−/−^ and CD300f^−/−^ mice were used as source of bone marrow-derived DCs. Briefly, 4 × 10^6^ marrow cells, free of red cells, were placed per 75 cm^2^ cell culture flask in 10 ml of medium RPMI-1640 complemented with 10% of heat-inactivated FCS, 100 U/ml penicillin, 100 *μ*g/ml streptomycin, 2 mM L-glutamine, 40 mM Hepes and 40 ng/ml rGM-CSF (Peprotech, Rocky Hill, NJ, USA). At day 2, another 10 ml of RPMI-1640 complete medium containing 40 ng/ml rGM-CSF was added to the flask. At days 4 and 6, half of the culture supernatant was removed, centrifuged and the cell pellet suspended in 10 ml of fresh completed medium with rGM-CSF was added back into the original flasks. Cells were used on day 7 of culture. DCs were incubated or not with infected (eGFP^hi^RFP^+^) and uninfected (eGFP^hi^RFP^−^) neutrophils for 12 or 16 h. In some experiments, infected and uninfected neutrophils were fixed or not with paraformaldehyde (2%) and washed three times with complete medium before culture with DCs, or were treated with anti-Mer antibody before neutrophil incubation. In brief, DCs were incubated with anti-Fc-*γ* III/II (CD16/32) receptor Ab (2.4G2, BD Biosciences) for 30 min and 20 *μ*g of goat anti-Mer antibody (AF591, R&D Systems, Minneapolis, MN, USA) or goat IgG (isotype control) for 1 h at 37 °C. Following co-culture, subpopulations of CD11c^+^GFP^-^ and CD11c^+^GFP^+^ DCs from the culture of DCs with infected or uninfected neutrophils, and CD11c^+^ DCs from control culture in the absence of neutrophils, were analyzed for the expression of activation markers or sorted, and then analyzed by confocal microscopy or further used as APCs. To compare the APC function of infected and uninfected DCs, these cells were incubated or not for 12 h with *Lm*-RFP metacyclic promastigotes opsonized by incubation for 30 min at 37 °C in 10% fresh normal mouse serum (1 parasite per DC). CD11c^+^ and CD11c^+^RFP^+^-infected DCs were also isolated by cell sorting and used as APCs. For analysis of the capacity of directly infected DCs and DCs infected via uptake of parasitized neutrophils to present parasite-derived antigen, DCs were incubated with opsonized *Lm*-NT-OVA-RFP metacyclic promastigotes or with sorted, infected (eGFP^hi^RFP^+^) neutrophils recovered from the ear dermis following injection of *Lm*-NT-OVA-RFP in a ratio of 1 parasite or 1 infected neutrophil per DC. After 16 h, CD11c^+^RFP^+^ directly infected DCs and CD11c^+^RFP^+^ DCs infected via uptake of parasitized neutrophils were sorted and used as APCs. The viability of sorted DCs following their engulfment of infected or uninfected neutrophils was assessed using Trypan blue and was routinely >98%.

### Purification of T lymphocytes and cell proliferation assays

CD8^+^ lymphocytes were purified from spleens of RAG1-deficient OT-I CD8^+^ TCR transgenic mice by negative selection (MACS system; Miltenyi Biotec, Auburn, CA, USA), according to the manufacturer's indications. Purified T cells were incubated at 2.5–5 × 10^7^ cells/ml in PBS with 0.5 *μ*M CFDA (Invitrogen, San Diego, CA, USA) for 10 min at 37 °C. The reaction was stopped with RPMI-1640+10% FCS, and the cells were washed twice with cold PBS+1% FCS. CFDA-labeled CD8^+^ T cells (3 × 10^4^–3 × 10^5^) were placed per well in 96-well round-bottom plates in 200 *μ*l of RPMI-1640 complete medium+10% FCS with 3 × 10^3^–3 × 10^4^ APCs (10 T cells per DC). Ovalbumin antigen (200 *μ*g/ml) or SIINFEKL peptide (1 nM) was added to the cultures, except experiments involving *Lm*-NT-OVA-RFP parasites, and incubated at 37C, 5% CO_2_ for 72 h. IFN-*γ* in culture supernatants was quantitated by ELISA, following the manufacturer's protocol (eBioscience, San Diego, CA, USA).

### Immunolabeling and flow cytometry

Bone marrow-derived DCs were incubated with an anti-Fc-*γ* III/II (CD16/32) receptor Ab (2.4G2, BD Biosciences) in RPMI without phenol red (Gibco) containing 1% FCS. The following antibodies were used: APC-anti-mouse CD11c (HL3, BD Biosciences), PE-Cy7-anti-mouse CD11c (N418, eBioscience), Alexafluor-700 anti-mouse MHC II (M5/114.15.2, eBioscience), APC-anti-mouse CD40 (1C10, eBioscience), APC-anti-mouse CD80 (16–10A1, eBioscience) and PerCP-Cy5.5-anti-mouse CD86 (GL-1, BioLegend, San Diego, CA, USA). The isotype controls used (all obtained from BD Biosciences) were rat IgG1 (R3-34) and rat IgG2b (A95-1). For intracellular detection of cytokines, single-cell suspensions from the skin were processed in the presence of GolgiPlug (BD Biosciences). Following surface staining with PE-Cy7-anti-mouse CD11b (M1/70, eBioscience), PerCP-Cy5.5-anti-mouse Ly6C (HK1.4, eBioscience) and FITC-anti-mouse Ly6G (1A8, eBioscience), cells were permeabilized and then stained with a combination of anti-mouse antibodies: Pac Blue anti-IL6 (MP5-20F3, eBioscience), Pac Blue anti-IL12p70 (C15.6, eBioscience), APC anti-IFN-*γ* (XMG1.2, eBioscience) and APC anti-TNF-*α* (TN3-19, eBioscience) in Perm/Wash buffer (BD Bioscience). Intracellular staining was carried out for 30 min on ice. For CD8^+^ T-cell proliferation analyses, the following antibodies were used: APC anti-CD8*α* (53-6.7, eBioscience), or PeCy7 anti-TCR*β* (H57-597, BioLegend). The data were collected and analyzed using FacsCANTO flow cytometer (BD Biosciences) and FlowJo software (TreeStar, Ashland, OR, USA), respectively.

### Confocal microscopy and image processing

Images were collected on a Leica SP5 inverted confocal microscope with a 63 × NA 1.4 oil immersion objective (Leica Microsystems, Buffalo Grove, IL, USA). Images were acquired with thin *z*-stack for 3D reconstruction using Imaris, Image analysis software (Bitplane Inc., South Windsor, CT, USA).

### Real-time PCR

For analysis of cytokine gene expression, sorted populations of neutrophils, recovered as described above, were placed in RNAlater (Qiagen, Los Angeles, CA, USA). Homogenates were then passed through QIAshredder columns, and RNA was purified using an RNeasy minikit according to the manufacturer's protocol (Qiagen). Reverse transcription was performed using the SuperScript III first-strand synthesis system for reverse transcription-PCR (RT-PCR) (Invitrogen Life Technologies, San Diego, CA, USA). Real-time PCR was performed on an ABI Prism 7900 sequence detection system (Applied Biosystems, Atlanta, GA, USA). Primer probe sets were from predeveloped gene expression assays designed by Applied Biosystems. The quantity of the product was determined by the comparative threshold cycle method using 2^−ΔΔ*CT*^ (where *C*_T_ represents the cycle threshold) to determine the fold increase. Each gene was normalized to the 18S rRNA endogenous control.

### Statistical analysis

Statistical significance between groups was determined by the unpaired, two-tailed Student's *t-*test using Prism software (GraphPad, La Jolla, CA, USA).

## Abbreviations

DC: dendritic cell
APC: antigen-presenting cell
RFP: red fluorescent protein
PtdSer: phosphatidylserine
Gas6: growth-arrest specific protein 6
MPO: myeloperoxidase
OVA: ovalbumin
i.d.: intradermal
