# Supplementary material for: Apoptotic cell clearance of Leishmania major-infected neutrophils by dendritic cells inhibits CD8+ T-cell priming in vitro by Mer tyrosine kinase-dependent signaling
Source: Cell Death Dis. 2015 Dec 10;6(12):e2018–. doi: 10.1038/cddis.2015.351 (PMC4720886; doi:10.1038/cddis.2015.351)
Supplement: Supplementary Information [file cddis2015351x1.pdf]

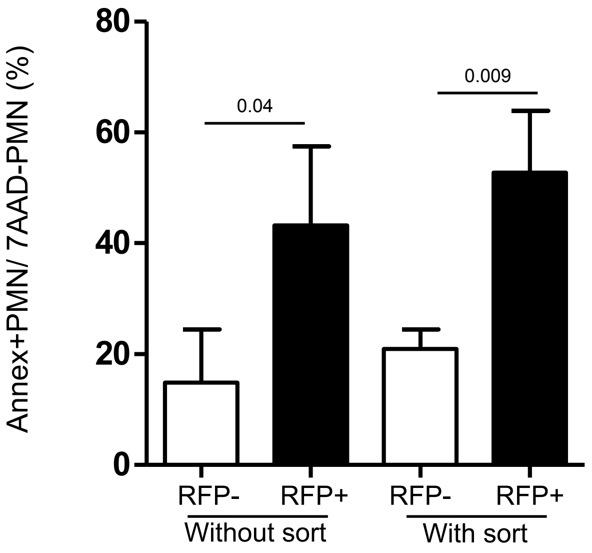

Figure S1. Frequencies of annexinV^{+} 7-AAD^{-} uninfected eGFP^{hi}RFP^{-} or infected eGFP^{hi}RFP^{+} neutrophils recovered from the ear dermis at 12 hrs after infection with 2 x10^{6} *Lm*-RFP metacyclic promastigotes purified or not by cell sorting. Mean percentage +/- 1 s.d., data are pooled from three independent experiments.

Supplementary figure 2

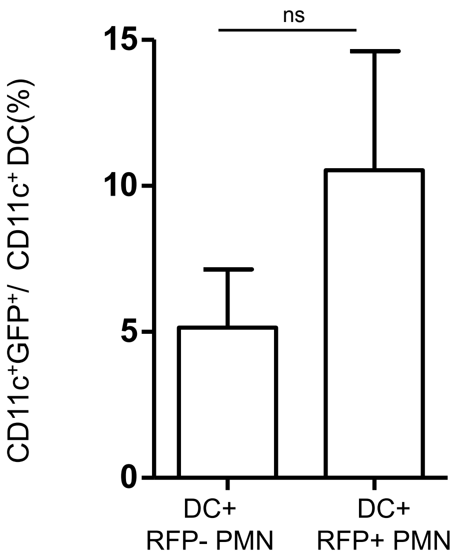

Figure S2. Frequencies of CD11c^{+}GFP^{+} DCs from culture with uninfected eGFP^{hi}RFP^{-} or infected eGFP^{hi}RFP^{+} neutrophils for 16 hrs (mean percentage +/- 1 s.d.) calculated from 3 independent experiments.

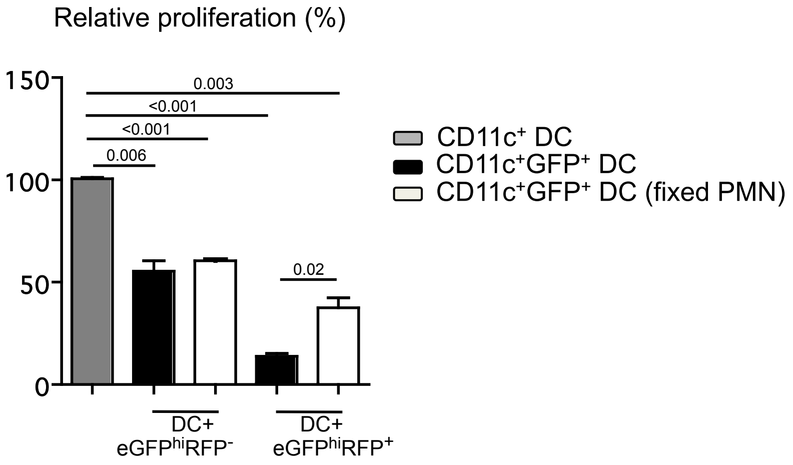

Figure S3. Relative proliferation of CFDA-labeled OT-I CD8^{+} T cultured for three days with sorted CD11c^{+} (no exposure to neutrophils), CD11c^{+}GFP^{-} and CD11c^{+}GFP^{+} cells (following overnight culture with fixed or unfixed uninfected or infected neutrophils), in presence of soluble OVA. Mean percentage +/- 1 s.d. calculated from 2 independent experiments.

Supplementary figure 4

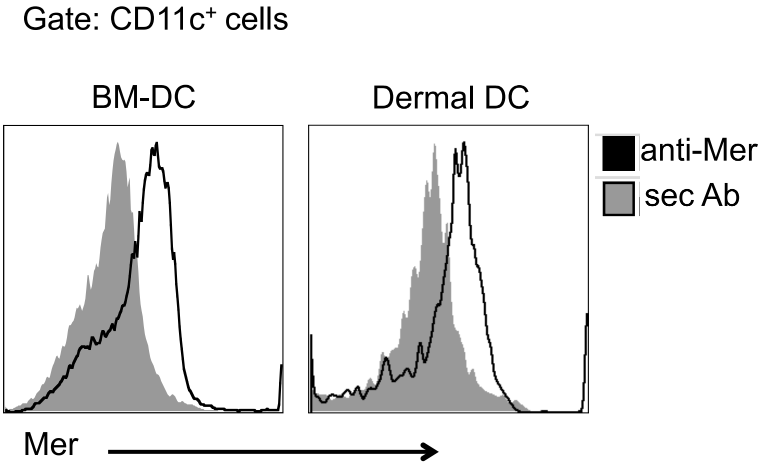

Figure S4. Mer expression on bone marrow derived-DCs and dermal DCs. Representative histogram plot of Mer expression on CD11c^{+} gated cells, anti-Mer (black line) and isotype control (gray filled) stained cells.

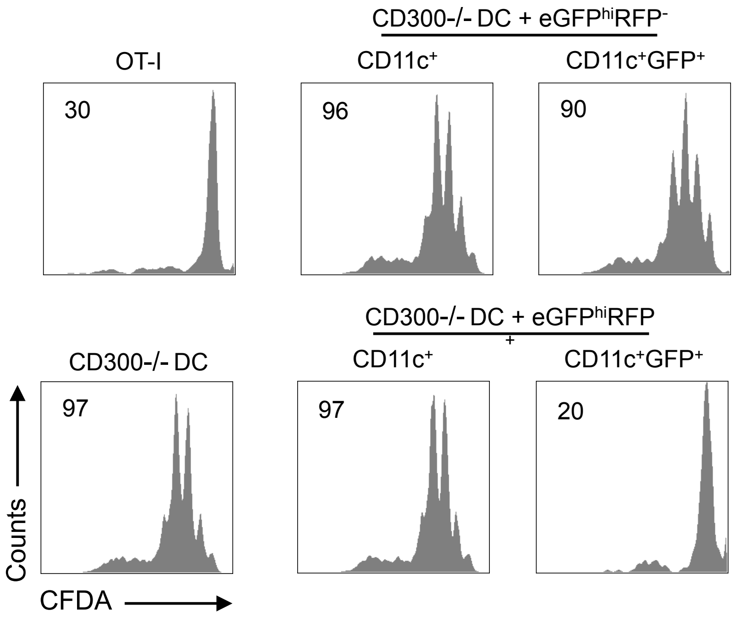

Figure S5. Antigen presentation function of CD300f-deficient DCs that have captured *L. major*-infected and uninfected neutrophils. CD300f $^{-/-}$ DCs were cultured or not with eGFP^{hi}RFP^{-} and eGFP^{hi}RFP^{+} dermal neutrophils for 12-16 hrs. Sorted CD11c^{+}, CD11c^{+}GFP^{-} and CD11c^{+}GFP^{+} cells were cultured with CFDA-labeled OT-I CD8^{+} T cells for three days, in presence of OVA antigen. Representative histogram plots of CFDA fluorescence of CD8^{+}CD3^{+} gated cells showing the proliferative response. Numbers represent the frequency of cells with reduced CFDA content.

Supplementary figure 6

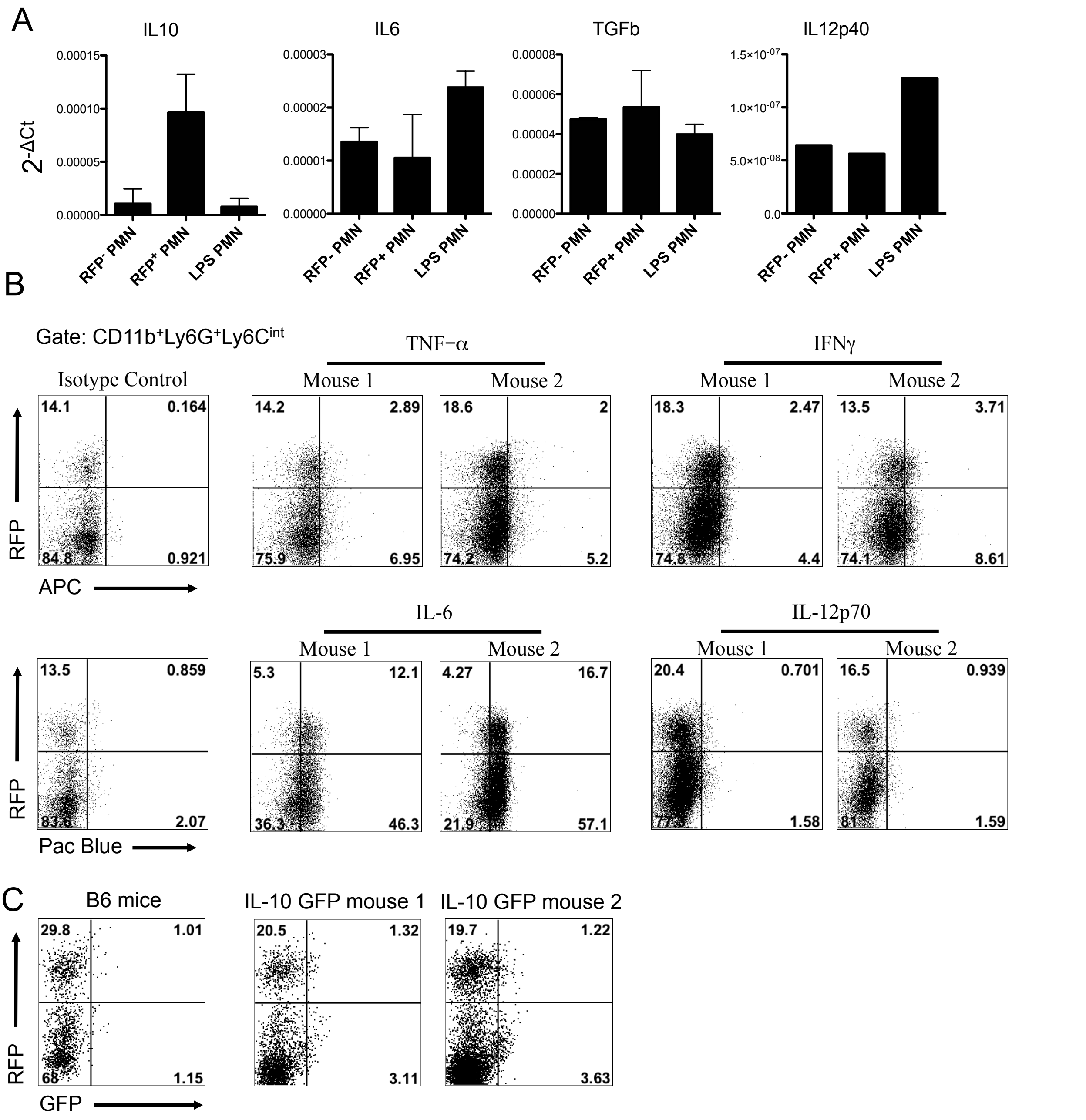

Figure S6. Cytokine expression in *L. major*-infected and uninfected neutrophils. (A) eGFP^{hi} neutrophils recovered from the ear dermis 12 hrs after infection with 2 x10^{6} *Lm*-RFP metacyclic promastigotes or injection of 10 ng of LPS were sorted to obtain uninfected eGFP^{hi}RFP^{-} and infected eGFP^{hi}RFP^{+} neutrophils, and in situ LPS stimulated neutrophils. Dermal neutrophils were immediately processed for isolation of RNA and cytokine gene expression was measured by real-time PCR. (B) CD11b^{+}Ly6G^{+}Ly6C^{int} dermal neutrophils recovered 12hrs after infection with 2 x10^{6} *Lm*-RFP metacyclic promastigotes were analyzed for the expression of RFP and intracellular cytokines. (C) CD11b^{+}Ly6G^{+}Ly6C^{int} neutrophils recovered 12 hrs after injection of 2 x10^{6} *Lm*-RFP metacyclic promastigotes into the ear dermis of IL-10 GFP mice were analyzed for the expression of RFP and GFP signal.
